# Supplementary material for: The impact of alcohol misuse in trauma patients: A scoping review protocol
Source: PLoS One. 2024 Oct 30;19(10):e0301241. doi: 10.1371/journal.pone.0301241 (PMC11524505; doi:10.1371/journal.pone.0301241)
Supplement: Attachment — Submitted filename: PlosOne Author Feedback- June22.docx [file pone.0301241.s002.docx]

**Abstract:**

- Please state the Open Science Framework study registration number.

**Introduction:**

- Providing a definition of unintentional injuries and traumatic injuries will help with reader comprehension.
- Line 74 states “no current or underway systematic reviews or scoping reviews on the topic were identified”. This is in a way contradictory as a scoping review is considered to be a type of systematic review as per JBI methodology. Please reword this line.

**Methods:**

- The method section is lacking as no scoping review principles are stated to be followed. To improve this section please state what scoping review principles you are following is it JBI, Cochraine, something else? Stating this and following the principles of your choice will help improve your manuscript.
- Line 84, please provide Open Science Framework registration link, please state the registration number instead.
- Under eligibility criteria, please clarify in the methods section the specific types of literature being included in the scoping review. Will you be including published, unpublished and/or grey literature. Subsequently, please provide rational for which you are using and which you are excluding.
- Under inclusion criteria:
  - On line 93-94 it is stated that studies from 2000 onward will be included with the justification “in keeping with similar evaluations of literature”. Please expand on this justification. Often scoping reviews do not restrict by dates, it is okay to do so but requires more justification.
  - Studies to be included are stated on line 95-97. Please consider including additional studies like mixed methodology or qualitative pieces as they could provide additional information about ETOH exposure at the event, more so than strictly quantitative studies could.
  - Line 94 states that included studies will be included if they are published in English. This is common however groups such as JBI are moving away from English only and encouraging authors to include studies of all languages. As general advice from this reviewer please consider using free software such as DeepL (<https://www.deepl.com/en/translator>) to be able to include non-English studies.
- Under exclusion criteria:
  - Please be explicit about where you are accessing acticles through, for example line 99 states “…full text articles that could not be accessed will be excluded” but where is access coming from, an institution?
  - Line 102-104 states studies with exclusive groups that will not be included in the scoping review. Two groups are referenced that may cause the reader some confusion, the exclusion of cohorts with psychological trauma and burn patients. Please justify the exclusion of these groups.
  - Line 106 states “patients who were not admitted to the hospital… will be excluded”. Please clarify if patients who died in the ED will be included or excluded in this study.
  - Table 1 is a great way to summarize inclusion and exclusion criteria for the reader.
    - In this table, under exclusion criteria TBI as only injury is excluded, but has the author considered what will be done with studies that report multiple cases where patients with TBI may be included. Please refine table to reflect if just studies with TBI only patients will be included or if they will be included in the before mentioned circumstances.
- Under outcome measures
  - Line 122 states “Demographics when available will be evaluated (mean, age, etc…)” please reword this line to improve understanding.
- Under search strategy
  - Later in the acknowledgements the authors state that a librarian was consulted. Please reference the consultation of a librarian under search strategy.
  - It may strengthen the scoping review to have the search strategy Peer Review of Electronic Search Strategies (PRESSED). Here an additional librarian peer reviews the search strategy. If this has not been done, please considering having the strategy PRESSED. If this was completed already, please state it explicitly.
  - Line 138-139 reference when the search will be completed. Please reword and remove mention of submission of the manuscript. Instead it would be sufficient to state the month and year the search was carried out.
- Under data extraction, evaluation and synthesis
  - Throughout this section “authors” are referenced, please change the word to reviewers.
  - It is great practice to pilot as the author referenced. However this section would be strengthened if the authors stated how many articles were piloted, instead of “ten percent of the full text articles”. This would be quite a lot depending on how many articles are yielded. I would encourage the authors to pick a number instead of a percentage to pilot. For example ten articles could be a sufficient pilot.
  - Line 164 references qualitative studies, however in previous section they are not mentioned as being included. Please clarify.
  - Line 165 states “few will summarize findings for each outcome reported by at least 2-3 papers in a narrative format”. Please provide rational for the 2-3 paper mention, or select a definitive number with justification.
  - Line 168 please edit from “lack of high quality” to “lack of high quality studies’.

**Discussion:**

- Line 178 references “strong evidence-based conclusions”. Please be aware than coming to definitive evidence based conclusion is not the purpose of a scoping review, instead it is a scan of the available literature. Please remove this reference.
- Line 179 please change “trend” to “trends”.

**Other:**

- In acknowledgements the use of a librarian is reference, as previously mentioned please address this consultation under search strategy.
- In acknowledgements the help of a research assistant is referenced for reviewing and editing the protocol article. Please reference authorship guidelines of PLoS One and reflect on Brenda’s contributions. Please consider if the research assistants contribution would be substantial enough to be a author on this paper.
- I would encourage the authors to reflect on their choice of a scoping review. As presented, with the above edits applied a scoping review would be appropriate for this research problem. However, throughout the manuscript at times it does read as if the authors really want to do a traditional scoping review without a meta analysis.
